# Solid Pseudopapillary Neoplasm of the Pancreas Showing Spontaneous Regression Followed by Regrowth: A Case Report

**DOI:** 10.70352/scrj.cr.26-0188

**Published:** 2026-06-10

**Authors:** Yuji Ikeda, Daisuke Hashimoto, So Yamaki, Kazuki Matsumura, Hiroyuki Ishida, Satoshi Mii, Hidetaka Miyazaki, Yuki Matsui, Sang Thanh Nguyen, Sohei Satoi

**Affiliations:** 1 Department of Pancreatobiliary Surgery, Kansai Medical University, Hirakata, Osaka, Japan; 2 Department of Surgery, Trung Vuong Hospital, Ho Chi Minh, Ho Chi Minh, Vietnam; 3 Division of Surgical Oncology, University of Colorado Anschutz Medical Campus, Aurora, CO, USA

**Keywords:** solid pseudopapillary neoplasm, pancreatic tumor, pancreatectomy, laparoscopic surgery, long-term follow up

## Abstract

**INTRODUCTION:**

Solid pseudopapillary neoplasm (SPN) of the pancreas is a rare, low-grade malignant tumor. Although SPNs generally exhibit indolent behavior, spontaneous regression detected on CT imaging is exceedingly rare, and to the best of our knowledge, regrowth following regression has not been previously reported.

**CASE PRESENTATION:**

A 33-year-old woman was referred to Kansai Medical University following the detection of liver dysfunction during a routine medical checkup. Imaging revealed a well-demarcated 20-mm mass in the pancreatic body, and an endoscopic US-guided biopsy confirmed SPN. Surgical resection was initially planned but postponed because of a COVID-19 infection in a fellow inpatient. During follow-up, the tumor showed spontaneous regression and became undetectable on imaging at 4 months. However, MRI at 16 months revealed tumor regrowth, which became clearly visible on T2-weighted imaging by 23 months, measuring 15 mm. At 24 months, a laparoscopic spleen-preserving distal pancreatectomy was performed. Histopathology confirmed SPN with intratumoral hemorrhage and characteristic pseudopapillary architecture. Complete resection was achieved, and the patient’s postoperative course was uneventful.

**CONCLUSIONS:**

This case illustrates that SPN can regress spontaneously, yet subsequently regrow, underscoring the need for careful long-term imaging surveillance even after apparent tumor shrinkage.

## Abbreviations

CECT
: contrast-enhanced CT
DWI
: diffusion-weighted imaging
EUS
: endoscopic ultrasonography
SPN
: solid pseudopapillary neoplasm

## INTRODUCTION

SPN was first reported by Frantz in 1959.^1)^ SPN is a low-grade malignant pancreatic neoplasm that predominantly affects young women. It accounts for approximately 0.17%–2.7% of all exocrine pancreatic tumors, making it a relatively rare condition.^2,3)^

Because of its rarity and generally indolent clinical course, the natural behavior of SPN has not been fully elucidated. Most reported cases have been treated surgically shortly after diagnosis, and information on the natural history of untreated SPN remains limited. As a result, the potential for spontaneous changes in tumor size during active surveillance is not well understood. To the best of our knowledge, no previous reports have described an SPN that exhibited initial regression followed by regrowth during active surveillance. We herein report such a rare case.

## CASE PRESENTATION

A 33-year-old woman was referred to Kansai Medical University following the detection of liver dysfunction during a routine medical checkup. CECT revealed a well-demarcated, hypovascular tumor measuring 20 mm in diameter in the pancreatic body (**Fig. 1A** and **1B**). The internal heterogeneity of the tumor was suggestive of intratumoral hemorrhage. MRI demonstrated a well-circumscribed mass with high signal intensity on T2-weighted images, along with slight dilatation of the distal pancreatic duct. DWI showed high signal intensity at the corresponding site (**Fig. 1C** and **1D**).

**Fig. 1:**
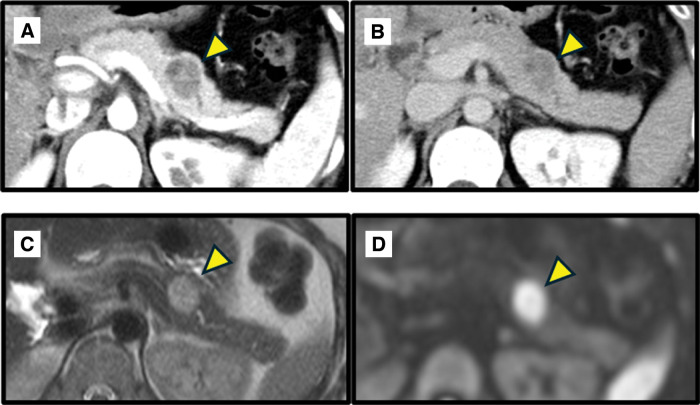
Imaging findings at initial diagnosis. Yellow arrowhead indicates the tumor. (**A**) CECT in the arterial phase showing a well-defined tumor. (**B**) CECT in the portal venous phase demonstrating the same lesion. (**C**) MRI T2-weighted image revealing a hyperintense tumor with dilatation of the pancreatic duct on the caudal side. (**D**) MRI diffusion-weighted image showing restricted diffusion of the tumor. CECT, contrast-enhanced CT

EUS demonstrated a well-defined hypervascular tumor with partial cystic change (**Fig. 2**), and an endoscopic US-guided biopsy was performed using a 22-gauge needle (SharkCore FNB Exchange System; Medtronic, North Haven, CT, USA) with 3 passes. No procedure-related complications were observed. Histological examination of the biopsy specimen showed positivity for β-catenin, progesterone receptor, and dot-like CD99 and negativity for INSM1 and E-cadherin, leading to the diagnosis of SPN. Laparoscopic spleen-preserving distal pancreatectomy was planned; however, the surgery was postponed because of a COVID-19 infection detected in another patient in the same ward.

**Fig. 2:**
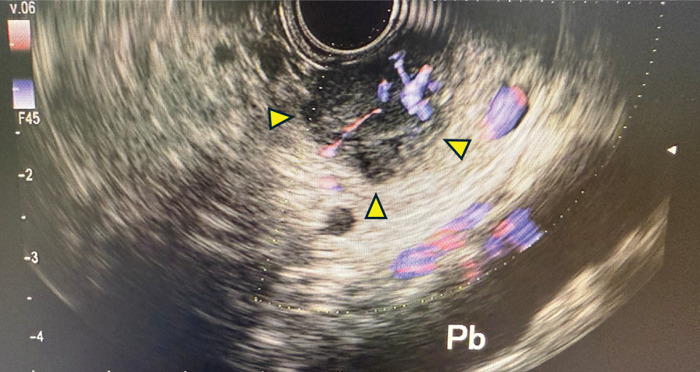
Endoscopic US. A well-circumscribed, heterogeneous hypoechoic mass is observed. A partial cystic component is present within the lesion, and abundant internal blood flow is present. (The yellow arrowheads indicate the hypoechoic mass.)

A CT scan performed 1 month after surgical postponement revealed that the tumor had decreased in size from 20 to 5 mm in diameter (**Fig. 3A**). After discussion with the patient, active surveillance was begun. Four months later, the tumor had become undetectable on CT imaging (**Fig. 3B**). Surveillance imaging was changed to MRI performed every 6 months to avoid continued radiation exposure.

**Fig. 3:**
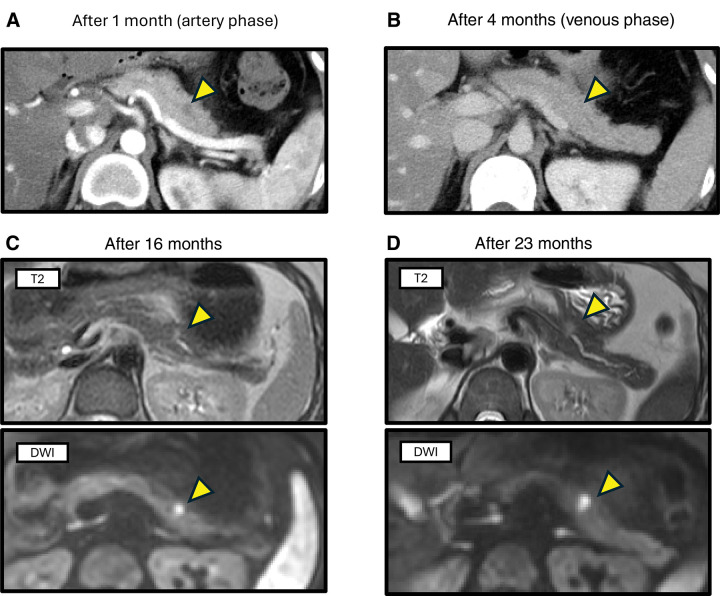
CT and MRI findings after surgical postponement. CT performed at 1 and 4 months after surgical postponement demonstrated progressive tumor shrinkage, and the tumor was no longer identifiable on the 4-month CT scan (**A** and **B**). At 16 months, DWI indicated regrowth of the tumor, although no obvious lesion was detected on T2-weighted imaging (**C**). At 23 months, MRI revealed an expanded hyperintense area on DWI, and the tumor became clearly visible on T2-weighted imaging (**D**). It was accompanied by dilatation of the pancreatic duct on the caudal side. (The yellow arrowhead indicates the tumor.) DWI, diffusion-weighted imaging

The 10-month MRI scan was negative for residual tumor. At 16 months, MRI-DWI revealed tumor regrowth measuring 5 mm in diameter (**Fig. 3C**). At 23 months, the lesion had further increased in size to 15 mm in diameter and became clearly visible on T2-weighted imaging (**Fig. 3D**).

After obtaining the patient’s consent, we performed surgery. A laparoscopic spleen-preserving distal pancreatectomy was performed 24 months after the initial active surveillance (**Fig. 4**). The operative time was 429 min with an estimated blood loss of 114 mL. The postoperative course was uneventful, and the patient was discharged on POD 11.

**Fig. 4:**
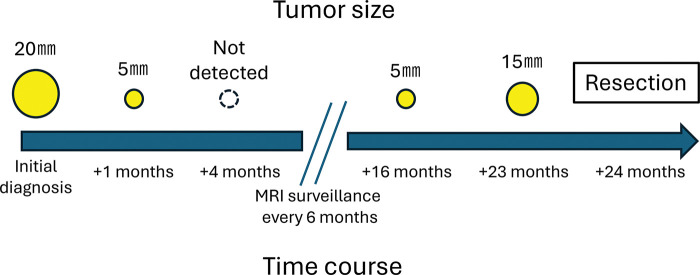
Timeline of changes in tumor size during surveillance. Tumor sizes and the timing of imaging examinations from the initial visit to surgery are shown.

Macroscopically, the resected specimen revealed a well-circumscribed, reddish-brown nodular lesion measuring 12 mm in diameter (**Fig. 5A**). Histologically, the solid tumor was composed of proliferating cells with round nuclei and eosinophilic cytoplasm, with pseudopapillary structures centered around the capillaries (**Fig. 5B** and **5C**). Hemorrhage and foamy macrophages were present within the lesion. The Ki-67 labeling index was <5%. Immunohistochemistry showed nuclear positivity for β-catenin expression and positivity for CD10 and vimentin expression. Chromogranin A expression was negative (**Fig. 5D**), whereas synaptophysin was focally positive (**Fig. 5E**). Estrogen receptor staining was negative, whereas progesterone receptor expression was positive. These findings were definitive for the diagnosis of SPN, and complete resection was confirmed. No cystic lesion corresponding to that identified on EUS was observed in the resected specimen. At 15 months after surgery, the patient remains free of recurrence or metastatic disease and continues to do well.

**Fig. 5:**
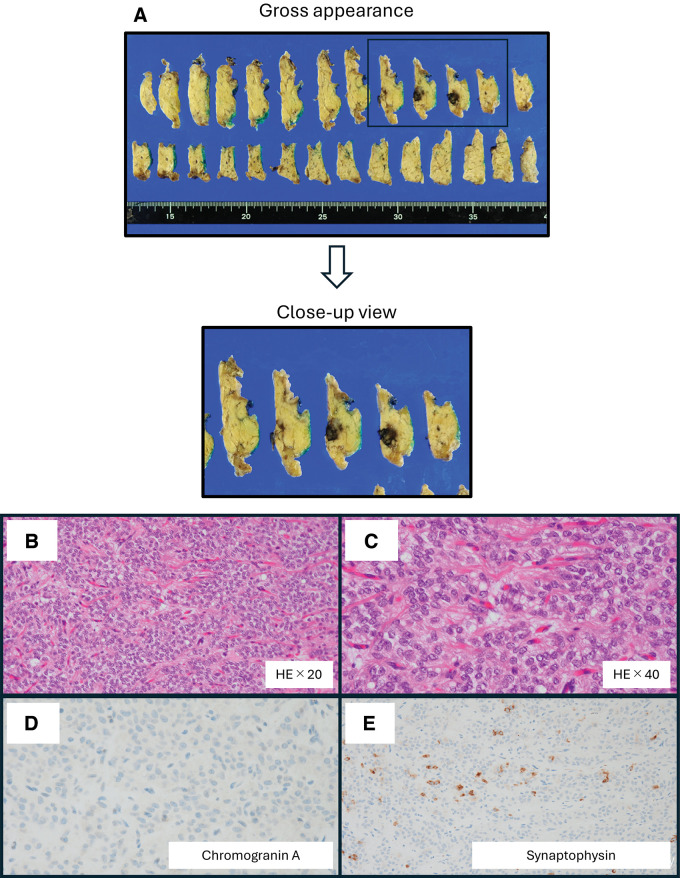
Gross and histopathological findings of the resected tumor. In the gross examination (**A**), the resected specimen shows a relatively well-circumscribed, reddish-brown nodular lesion (close-up view). Histopathologically, HE staining at low magnification (×20) (**B**) and high magnification (×40) (**C**) demonstrates solid proliferation of tumor cells with round to oval nuclei and eosinophilic cytoplasm. Pseudopapillary structures centered on delicate capillaries are also observed. Hemorrhage and clusters of foamy macrophages are present within the tumor. Immunohistochemically, the tumor cells are positive for nuclear β-catenin, CD10, and vimentin expression. The tumor cells are negative for chromogranin A expression, as shown in (**D**), whereas the cells are partially positive for synaptophysin expression, as shown in (**E**). HE, hematoxylin-eosin

## DISCUSSION

SPN has been increasingly diagnosed because of advances in imaging technology.^4)^ It typically arises in the pancreatic body or tail and appears as a well-circumscribed lesion that is often associated with cystic degeneration or intratumoral hemorrhage.^5–7)^ A large-scale review of 2450 Chinese patients supports these general characteristics, demonstrating a strong female predominance and overall favorable postoperative prognosis. Because the review was based on heterogeneous retrospective reports, it is best interpreted as epidemiological background information.^8)^

Distant metastasis is uncommon, and although tumor size, invasive features, and the Ki-67 index have been suggested as indicators of malignant potential, no definitive criteria have been established.^9–14)^ Surgical resection remains the standard treatment for SPN and is associated with favorable long-term outcomes.^6)^

Multivariate analysis has shown that tumor size >8 cm, microscopic malignant features, and stage IV disease are significant prognostic factors for recurrence after resection of the tumor, indicating that the risk of recurrence is primarily determined by tumor size and histopathological aggressiveness.^15)^

Although the efficacy of chemotherapy for unresectable SPN remains uncertain, tumor shrinkage has been reported in some cases, particularly with regimens including cisplatin.^16,17)^ In our case, the tumor was small, and the Ki-67 index was less than 5%, supporting its classification as a low-grade malignant tumor. Macroscopic discoloration on the cut surface corresponded to intratumoral hemorrhage and may explain part of the imaging variability observed during follow-up. SPN often expresses sex hormone receptors, and estrogen-related proliferative activity has been reported; however, standardized hormone-based therapy has not been established.^18)^

Our case is unique in that the tumor showed spontaneous regression and became radiologically undetectable, followed by subsequent regrowth. Several cases of spontaneous regression have been reported, predominantly in pediatric patients and premenopausal women.

Yasuda et al. reported spontaneous regression of a 3.0 × 1.9 cm SPN within 1 year after detection in a 48-year-old woman, while Nakahara et al. described a gradual reduction in tumor size from 4.5 to 1.0 cm over a 10-year period in an 18-year-old woman.^19,20)^ Suzuki et al. reported rapid regression of a 5.1-cm SPN in a 13-year-old boy following tumor hemorrhage caused by abdominal trauma, with the lesion becoming radiologically undetectable after 6 years of surveillance.^21)^ Hachiya et al. described 2 pediatric cases in which the tumors became undetectable within 12 and 18 months.^22)^

Yoon and Lim reported a remarkable case of a large SPN with liver metastases that regressed spontaneously over 13 years following portal vein embolization alone.^23)^ Only our case and the case reported by Yoon and Lim were histopathologically confirmed as SPNs. In our case, the diagnosis was established by EUS-guided fine-needle aspiration, whereas Yoon and Lim obtained pathological confirmation through US-guided percutaneous biopsy of the pancreatic mass. The tumor in Yoon and Lim’s case was treated by portal vein embolization; none of the other reported tumors were resected, and all were managed with observation alone.

Previous reports have suggested that spontaneous regression of SPNs may result from degenerative changes, including intratumoral hemorrhage, necrosis, cystic degeneration, and subsequent resorption, leading to an apparent reduction in tumor size.^19–23)^ However, these reports have described tumor shrinkage or disappearance alone, without documentation of subsequent regrowth. To our knowledge, this is the first report to demonstrate spontaneous regression with the lesion becoming radiologically undetectable, followed by subsequent tumor regrowth during active surveillance. In our case, intratumoral hemorrhage was histologically confirmed, supporting the hypothesis that degenerative changes contributed to the initial regression of the tumor. Another possible explanation is the effect of EUS-guided fine-needle aspiration. Temporary hemorrhage caused by the biopsy, rather than spontaneous intratumoral hemorrhage, may have been resorbed and subsequently recognized as tumor shrinkage. However, this alone is unlikely to explain why the lesion became radiologically undetectable. Although true tumor growth due to persistence of viable tumor cells cannot be entirely excluded, this possibility appears less likely given a Ki-67 labeling index of <5%. Therefore, the changes in tumor size observed in the present case were more likely attributable to intratumoral hemorrhage or cystic degeneration than to true tumor proliferation.

## CONCLUSIONS

In conclusion, spontaneous regression of SPNs does not necessarily indicate resolution of the tumor. Given the unpredictable natural history of SPN, with its potential for regrowth even after apparent radiological disappearance, surgical resection remains the definitive treatment, and careful long-term imaging surveillance is warranted.

## DECLARATIONS

### Availability of data and materials

All data generated or analyzed during this study are included in this published article.
